# CircRNAs as Novel Biomarkers and Therapeutic Targets in Renal Cell Carcinoma

**DOI:** 10.3389/fmolb.2022.833079

**Published:** 2022-02-11

**Authors:** Yuxia Zhou, Cheng Li, Zhenping Wang, Shuangfeng Tan, Yiqi Liu, Hu Zhang, Xuefeng Li

**Affiliations:** ^1^ The First Affiliated Hospital, Department of Medical Oncology, Hengyang Medical School, University of South China, Hengyang, China; ^2^ The Second Affiliated Hospital, Department of Urology, Hengyang Medical School, University of South China, Hengyang, China; ^3^ The First Affiliated Hospital, Department of Urology, Hengyang Medical School, University of South China, Hengyang, China

**Keywords:** CircRNAs, renal cell carcinoma, biomarker, targeted therapy, ceRNA

## Abstract

Circular RNAs (circRNAs) are a type of long non-coding RNA with covalently closed loops that are naturally resistant to exoribonuclease. With the rapid development of high-throughput sequencing technologies and bioinformatics, increasing data suggest that circRNAs are abnormally expressed in renal cell carcinoma (RCC) and act as important regulators of RCC carcinogenesis and progression. CircRNAs play important biological roles in modulating cell proliferation, migration, invasion, apoptosis, and gemcitabine chemoresistance in RCC. Most of the circRNAs studied in RCC have been reported to be significantly associated with many clinicopathologic characteristics and survival parameters of RCC. The stability and specificity of circRNAs enable them potential molecular markers for RCC diagnosis and prognosis. Moreover, circRNAs have emerged as targets for developing new therapies, because they can regulate various signaling pathways associated with RCC initiation and progression. In this review, we briefly summarize the biogenesis, degradation, and biological functions of circRNAs as well as the potential clinical applications of these molecules for RCC diagnosis, prognosis, and targeted therapy.

## Introduction

Renal cell carcinoma (RCC), the most common kidney neoplasm, originates in renal tubular epithelial cells and affects over 430,000 individuals all over the world per year (Sung et al., 2021). The incidence of RCC is increasing at a rate of about 2% year by year, growing the third most common urogenital malignancy (Znaor et al., 2015). Clear cell renal cell carcinoma (ccRCC), the most common and the most aggressive form of kidney cancer, accounts for up to 80% of the RCC new cases with high mortality. The other subtypes include papillary, chromophobe RCC, and collecting-duct carcinoma. Because of its high rates of metastasis and difficult diagnosis, poor prognosis is a typical feature of RCC. Up to 60% of RCC are detected by chance, due to the lack of obvious symptoms in the early stage (Petejova and Martinek, 2016). Nowadays, surgical resection is the first-line treatment and important intervention for local RCC. Unfortunately, about 30% of patients will develop local recurrence or relapse in distant sites even after radical nephrectomy (Ahrens et al., 2019). Moreover, most RCC patients are resistant to both chemotherapy and radiotherapy once the recurrence and metastasis occurred (Lara and Evans, 2019). The overall prognosis of patients with metastatic RCC is very poor and the 5-year survival rate at diagnosis is less than 10%, especially those high-stage patients. Over the past decade, the rise of kinase and immune checkpoint inhibitors has expanded therapeutic options available and brought great prospects for the treatment of RCC. Targeted therapies targeting vascular endothelial growth factor (VEGF) and mammalian target of rapamycin (mTOR) pathways have been developed, but these treatments are still palliative with limited effectiveness and most patients eventually suffer a relapse (Motzer et al., 2013; Motzer et al., 2014). Hence, it is necessary to explore the molecular mechanism of RCC and develop new targeted drugs.

Gene mutation and epigenetic disorder play an important role in the occurrence and development of RCC. It is well known that the von Hippel-Lindau (VHL) gene, one of the most common tumor suppressor mutated genes in RCC, is frequently inactivated in over 80% of ccRCC patients (Zhai et al., 2017). This leads to a blockage in the degradation of the α subunit of hypoxia-inducible transcription factors (HIF1 and HIF2) (Gossage et al., 2015), which results in increased expression of angiogenic factors including VEGF that plays a significant role in the growth and progression of RCC (Gudas et al., 2014). The phosphoinositide 3-kinase (PI3K)/AKT/mTOR pathway, which plays a crucial role in regulating cell growth, has also been shown to be constitutively activated in RCC (Elfiky et al., 2011). In recent years, the epigenetic changes of RCC, including non-coding RNA (ncRNAs), have become one of the research hotspots. The discovery of ncRNAs contributes to further understanding of the pathogenesis and treatment of RCC.

CircRNAs, a novel subclass of non-coding RNAs, are characterized with the covalently closed structure without 5′caps and 3′poly(A) tails. Due to the absence of free ends, circRNAs are naturally resistant to exoribonuclease and more stable than linear RNA both inside cells and in extracellular plasma, including blood, saliva, urine, and exosomes (Li et al., 2015a; Bahn et al., 2015; Zhang et al., 2017; Necula et al., 2019). Moreover, circRNAs have organ and tissue specific expression patterns, which have inspired numerous studies on their application as promising biomarkers of cancer (Kristensen et al., 2021). Unfortunately, circRNAs were initially regarded as splicing errors, and their important roles in gene regulation seem to be overlooked. In 1976, The existence of circRNAs was first reported in RNA viruses, like plant viruses (Sanger et al., 1976). A few years later, circRNAs were observed in the cytoplasm of eukaryotic cells (Arnberg et al., 1980). Until circular transcripts were detected in the testes of adult mice with sex-determining region Y genes, circRNAs were recognized to possess potent function (Dolci et al., 1997). In 2012, the abundance and ubiquity of circRNAs in eukaryotes were identified with the development of RNA high-throughput sequencing and novel computational approaches for non-polyadenylated RNA transcripts. Moreover, a report that ciRS-7 (also known as CDR1as) could regulate the gene expression serving as the microRNA (miRNA) sponge, initiated a burst in the research field of circRNAs (Hansen et al., 2013). In recent years, it has been demonstrated that numerous circRNAs are dysregulated and have been identified as important regulators of multiple diseases, such as cancers and cardiovascular diseases (Okholm et al., 2017; Zhou and Yu, 2017; Song and Li, 2018). It has been demonstrated that aberrant circRNA expression is common in cancer and they involved in the regulation of tumorigenic behaviors such as apoptosis, invasion, migration, and proliferation (Rajappa et al., 2020). In this review, we briefly summarize the biogenesis, degradation, and functions of circRNAs and elucidate circRNAs as novel biomarkers and therapeutic targets in RCC.

## Biological Characterization of CircRNAs

### Biogenesis of CircRNAs

**FIGURE 1:**
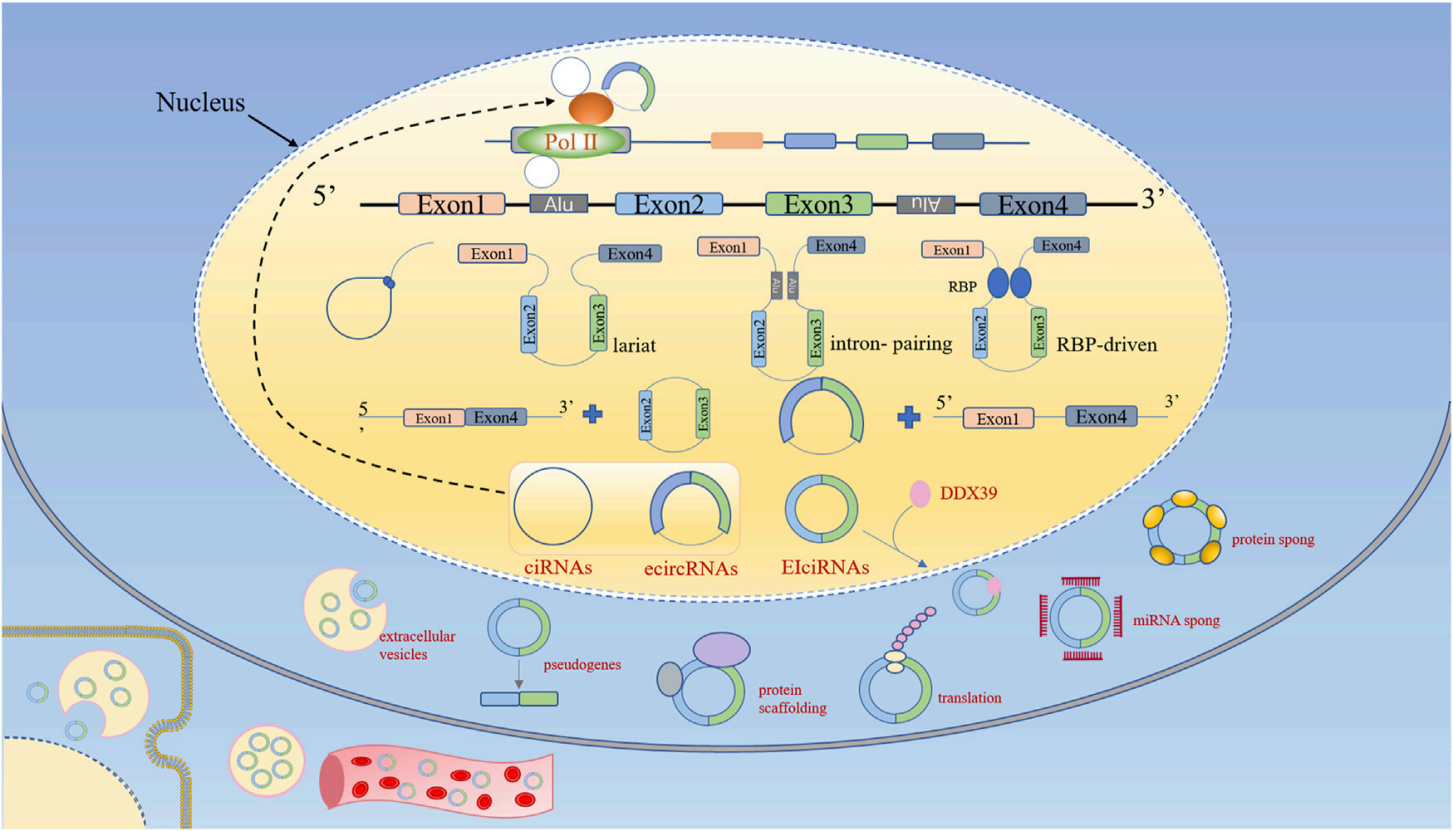
Biogenesis and functions of circular RNAs (circRNAs). According to their composition, circRNAs can be classified into four categories: ecircRNAs, ciRNAs, EIciRNAs, and tricRNAs. CircRNA biogenesis competes with linear pre-mRNA splicing. CircRNAs enhance the transcription and splicing of their parental genes by interacting with RNA pol II or U1 small nuclear snRNP. CircRNAs bind to RBPs to regulate their biological activity. CircRNAs function as miRNA sponges. CircRNAs can be translated into proteins. CircRNAs function as protein scaffolds. CircRNAs-derived pseudogenes. CircRNAs are eliminated by extracellular vesicles into extracellular space, and can be absorbed by other cells or could enter the circulation.

Precursor messenger RNA (pre-mRNA) is canonically spliced into functional linear RNA transcript with 5′ to 3′ polarity via removing introns. However, circRNAs are generally generated by back-splicing of precursor mRNAs(pre-mRNAs), a process of binding between the downstream 5′ splice donor site and upstream 3′ splice acceptor (Zhang et al., 2016a). According to their composition, circRNAs can be classified into four categories: exonic circRNAs (ecircRNAs); circular intronic RNAs (ciRNAs); exon–intron circRNAs (EIciRNAs); and transfer RNA (tRNA) intronic circRNAs (tricRNAs). Note that tricRNAs are formed by precursor tRNA. Three hypothetical models for circRNAs biogenesis mechanisms have been widely accepted (Figure 1): lariat-driven circularization; pairing-driven circularization; RBP-mediated circularization. The first model is that the nonadjacent exons are pulled closer due to the partial folding of RNA, then a downstream 5 splice site of an exon joins an upstream 3 splice site resulting in exon skipping. After that, the introns are removed to form ecircRNAs or EIcirRNAs (Jeck et al., 2013). The generation of ciRNAs is a special situation in the lariat-driven model, where intronic lariats escape from debranching (Zhang et al., 2013). Pairing-driven circularization can be mediated by base pairing in the exons between inverted repeat elements (such as Alu elements) (Ivanov et al., 2015; Kelly et al., 2015), but sometimes from non-repetitive complementary sequences (Zhang et al., 2014). The third model is that RNA binding proteins (RBPs) bind both sides of flanking intron sequences, and bring splice donors and splice acceptors sufficiently close with the RBPs (Such as Quaking (QKI) (Conn et al., 2015), and FUS (Errichelli et al., 2017)) attracting to each other, resulting in a bridge formed between the introns.

Emerging data have identified that circRNAs formation is modulated by cis-regulatory elements and trans-acting factors. Several recent studies have suggested that the processing of circRNAs can be accelerated by either RNA pairing of reversely complementary sequences across their flanking introns (Jeck et al., 2013; Liang and Wilusz, 2014; Zhang et al., 2014; Ivanov et al., 2015) or protein factors binding to pre-mRNAs to bridge flanking introns together (Ashwal-Fluss et al., 2014; Conn et al., 2015). For example, the immune factors NF90 and NF110 can promote the production of circRNAs by stabilizing intronic RNA pairs (Li et al., 2017a; Legnini et al., 2017). However, some RBPs suppress the biogenesis of circRNAs, such as adenosine deaminase acting on RNA 1 (ADAR1) (Ivanov et al., 2015) and ATP- dependent RNA helicase A (also known as DHX9) (Aktaş et al., 2017). In addition, the biogenesis of circular RNA is also affected by epigenetic changes within histones and gene bodies (Shukla et al., 2011; Bentley, 2014), heteronuclear ribonucleoprotein L (hnRNP), and SR protein (Kramer et al., 2015; Fei et al., 2017). In conclusion, evidence indicates that the regulation of circRNAs biogenesis is strictly regulated by different cis-elements and trans factors in cells, but the detailed mechanism needs to be further explored.

### Degradation of CircRNAs

Up to now, the elimination and degradation of circRNAs in cells remain poorly understood. Most of the exon-containing circRNAs are transported from the nucleus to the cytoplasm in a size-dependent manner through ATP-dependent RNA helicase ddx39 (Huang et al., 2018). Because circRNAs lack 5′ and 3′ ends, they are inherently resistant to the major enzymes of mRNA degradation (Ghosh and Jacobson, 2010). There is evidence that circRNAs are enriched and stable in exosomes and can be detected in blood and urine (Yan et al., 2017; Wang et al., 2019). For example, ciRS-7 and circHIPK3 are enriched in extracellular vesicles, which may be eliminated by extracellular vesicles and further removed by the reticuloendothelial system or secreted by the liver and the kidney (Li et al., 2015a; Lasda and Parker, 2016). Recently, RNase L, a secreted circRNAs endonuclease, has been identified to globally degrade circRNAs upon poly (I:C) stimulation or viral infection (Liu et al., 2019). Moreover, it has been demonstrated that a subset of circRNAs containing m6A is preferentially downregulated by RNase P/MRP (endoribonucleases) (Park et al., 2019). In addition, the Argonaute2 (AGO2) protein may involve in the cleavage of specific circRNAs by binding to miRNAs. For example, the degradation of CDR1as dependent on Ago2-mediated cleavage mediated by miR-671 (Hansen et al., 2011). It needs to mention that, ciRS-7 and ciRS-122 in exosomes can maintain their circular properties, and extracellular vesicles can be absorbed by other cells, and eventually function upon release in recipient cells, suggesting that excreted circRNAs may be involved in signaling pathways (Wang et al., 2020a).

### Functions of CircRNAs

The potential functions and biological activities of circRNAs have been extensively studied. The main functions of circRNAs include modulating the parental genes expression, regulating gene transcription, acting as miRNA sponges, translating into proteins or peptides, serving as protein scaffolding and reservoir, forming pseudogenes, and acting as biomarkers (Figure 1).

#### CircRNAs Can Modulate Parental Gene Expression

CircRNAs play an important role in regulating parental genes by interacting with proteins and RBPs. On the one hand, certain circRNAs containing introns, both EIciRNAs and ciRNAs, can interact with RBP and affect the mRNA expression of parental genes. For example, EIciRNAs interact with U1 small nuclear ribonucleoprotein (snRNP) to further combine with polymerase II (Pol II) and regulate the promoter region of host gene transcription (Li et al., 2015b). Additionally, ciRNAs and Pol II complex can directly interact to regulate parental gene transcription (Dolci et al., 1997; Li et al., 2015b). On the other hand, in the process of circRNAs formation, competitive complementary pairing between introns can reach a balance with linear RNA, which can affect mRNA expression and even protein translation. For example, circMBL impacts linear RNA formation, and circPABPN1 can inhibit PABPN1 mRNA translation through competing with PABPN1 mRNA for binding to the HuR protein (Abdelmohsen et al., 2017). Moreover, due to the lack of a translation start site, EcircRNAs play important roles in the regulation of protein expression by inducing translation failure as mRNA traps (Jeck and Sharpless, 2014).

#### CircRNAs as miRNA Sponges

Among its biological functions, circRNAs mainly exert their function as a miRNAs sponge. By binding to the 3′UTR of mRNAs, miRNAs affect the stability of mRNAs and modulate gene expression in the nucleus and cytoplasm (Salmena et al., 2011; Salmanidis et al., 2014). There is increasing evidence that quite a few of circRNAs could serve as competitive endogenous RNA (ceRNAs) or miRNAs sponge via binging with miRNAs through miRNA response elements (MREs) to downregulate their functions. Thus, circRNAs may indirectly regulate the translation of mRNAs, and exert a post-transcriptional regulatory effect on the target mRNAs (Bahn et al., 2015). CDR1as was the first miRNA sponge reported to negatively regulate miR-7, which was found to be highly and stably expressed in several cancers and the mammalian brain (Hansen et al., 2013; Yu et al., 2016; Piwecka et al., 2017; Tang et al., 2017). Research revealed that CDR1as contains over 70 binding sites for miR-7 and acts as a miR-7 sponge, where it increases the translation of the miR-7 targeted mRNA (Hansen et al., 2013). Of note, circRNAs can regulate the activity of multiple miRNAs. For example, circHIPK3 can suppress the activity of miR-558 (Li et al., 2017b), miR-124-3p (Chen et al., 2020a), and miR-7 (Zeng et al., 2018) under certain conditions. Although circRNAs functioning as molecular sponges has been widely reported, a recent study proved that most circular RNAs could not function as “bona fide” miRNA sponges (Militello et al., 2017).

#### CircRNAs Can Be Translated Into Proteins or Peptides

Though circRNAs do not contain a 5′ methylguanosine (m7G) cap structure and a poly (A) tail, several studies have shown that engineered circRNAs can encode proteins when they contain internal ribosome entry sites (IRESs) that can promote direct binding of initiation factors or the ribosome to the translatable circRNAs (Chen and Sarnow, 1995; Wang and Wang, 2015; Li et al., 2017a). Moreover, it has been demonstrated that circMBL, circZNF609, and circPINTexon2, containing an internal ribosome entry site, were able to encode a protein in a cap-independent manner (Legnini et al., 2017; Pamudurti et al., 2017; Zhang et al., 2018). Another mechanism of circRNAs translation involves the modification of N6-methyladenosine (m6A). Modification of m6A RNA in the 5′ untranslated region (UTR) promotes the efficient initiation of translation from circRNAs in human cells (Zhou et al., 2015; Yang et al., 2017). Additionally, the circRNAs with infinite small open reading frames (ORFs) can encode a functional peptide by rolling loop amplification in an IRES -independent manner (Abe et al., 2013). Nevertheless, it remains to be investigated whether these proteins or peptides formed from circRNAs have important functions.

#### Protein Scaffolding and Reservoir

CircRNAs maybe serve as protein scaffolding to provide binding sites for the assembly of multiple proteins, such as enzymes and their substrates, thus forming large protein complexes (Jeck and Sharpless, 2014; Hansen et al., 2016). For example, the interaction of circ-Foxo3 with MDM2 and p53 could promote MDM2-induced p53 ubiquitination and subsequent degradation (Du et al., 2017a). CircACC1 works with AMPK β and γ regulatory subunits to form ternary complexes to promote the activity of AMPK holoenzyme (Li et al., 2019). In addition to promoting chemical reactions, circRNAs can also block the protein function as protein scaffolds. For example, circFOXO3 can interact with both cell cycle proteins cyclin-dependent kinase 2 (CDK2) and p21 and prevents CDK2 from interacting with cyclin A and E, which arrest cell cycle progression (Du et al., 2016). circFOXO3 has been found to promote cardiac-cell senescence by interacting the senescence-related proteins ID-1 and E2F1 with the stress-related proteins focal adhesion kinase (FAK) and hypoxia-inducible factor 1α (HIF1α) in the cytoplasm, thereby restraining their function through preventing FAK localization to mitochondria or HIF1α translocation to the nucleus in stressed cells (Du et al., 2017b). An earlier study revealed that circRNAs could be used as a molecular reservoir (Li et al., 2017a). CircRNAs can form short and incomplete intramolecular double-stranded RNAs (dsRNAs) to bind NF90, NF110, and interferon-induced, dsRNA-activated protein kinase (PKR). Once the virus is infected, the abundance of circRNAs is significantly reduced, resulting in the release of NF90, and NF110, which promotes antiviral immune response (Li et al., 2017a; Liu et al., 2019).

#### CircRNAs-Derived Pseudogenes

A pseudogene is defined as any genomic sequence that contains a defective copy of a gene, which is similar to another gene and has no capacity for coding protein due to the accumulation of mutations (Vanin, 1985). In 2016, The pseudogenes that originated from circRNAs in the mammalian genome were first demonstrated. Soon after, researchers found the circSATB1-derived pseudogene region can specifically bind to CCCTC-binding factor, which could reshape chromosome configuration, suggesting that this circRNAs-derived can regulate gene expression. In addition, retrotransposed circRNAs can be inserted into the genome to alter the genome structure, interrupt host genome integrity and the potential for gene regulation (Dong et al., 2016). This finding may indicate a novel function of circRNAs, that is, circRNAs can change the composition of genomic DNA by inserting its retrotranscription product.

## CircRNAs in Renal Carcinoma

More and more studies have shown that circRNAs play pivotal roles in the occurrence and development of cancer and might function as cancer biomarkers and novel therapeutic targets (Song et al., 2018; Altesha et al., 2019; Arnaiz et al., 2019). A result from The Cancer Genome Atlas and Gene Expression Omnibus database showed that a total of 114 circRNAs were found to be associated with tumor initiation, progression, and metastasis after the intersection in ccRCC (Wei et al., 2020). The aberrant circRNAs commonly exist in RCC, playing the oncogenic or suppressive role and regulating cellular functions. In particular, dysregulated circRNAs are correlated with clinicopathological features, prognosis and survival in RCC patients, suggesting that these stable circRNAs can be promising biomarkers for cancer diagnosis and prognosis. What’s more, circRNAs play crucial regulatory roles in upstream of various signaling pathways related to RCC carcinogenesis and progression, which makes them attractive therapeutic targets for RCC.

### CircRNAs Profiles in RCC

Early detection of circRNAs was rare and limited because of the stable circular structure. With the development of detecting techniques and bioinformatics tools, investigations on circRNAs have been significantly increased and substantial progress has been made in the identification of differential expression of circRNAs in RCC. Genome-wide detection plays an essential role in circRNAs detection, and RNA sequencing (RNA-seq) was the first technology used for the detection of circRNAs genomes. A few years ago, a study using circRNAs microarrays found that 542 circRNAs were abnormally expressed in ccRCC, of which 324 circRNAs were significantly down-regulated, while 218 circRNAs were up-regulated in ccRCC tumors (Ma et al., 2020). In 2019, analysis of 7 matched ccRCC samples using the ArrayStar microarray approach showed that 78 up-regulated and 91 down-regulated circRNAs had more than 2-fold differences compared with adjacent normal tissue samples (Franz et al., 2019). Moreover, the transcriptome data downloaded from the Gene Expression Omnibus dataset, showed that 961 circRNAs were differentially expressed between ccRCC and normal tissues, and 255 circRNAs were differentially expressed between metastatic ccRCC and primary tumor tissues in total (Wei et al., 2020). These high-throughput results strongly suggest the important roles of these circRNAs in RCC development and progression.

### CircRNAs in RCC and Molecular Mechanisms

**FIGURE 2:**
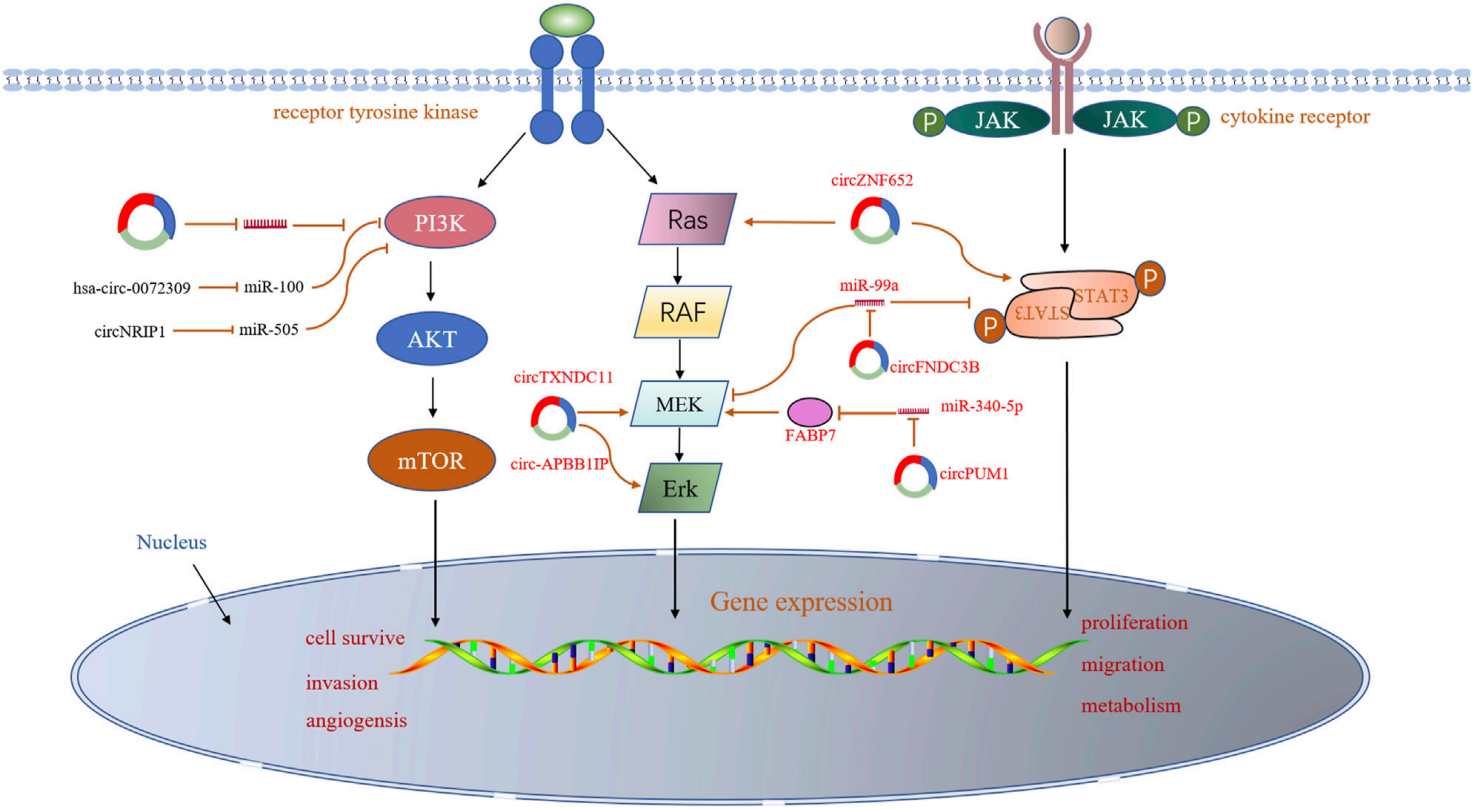
Involvement of circRNAs in the cancer-associated signaling pathways in RCC.

**FIGURE 3:**
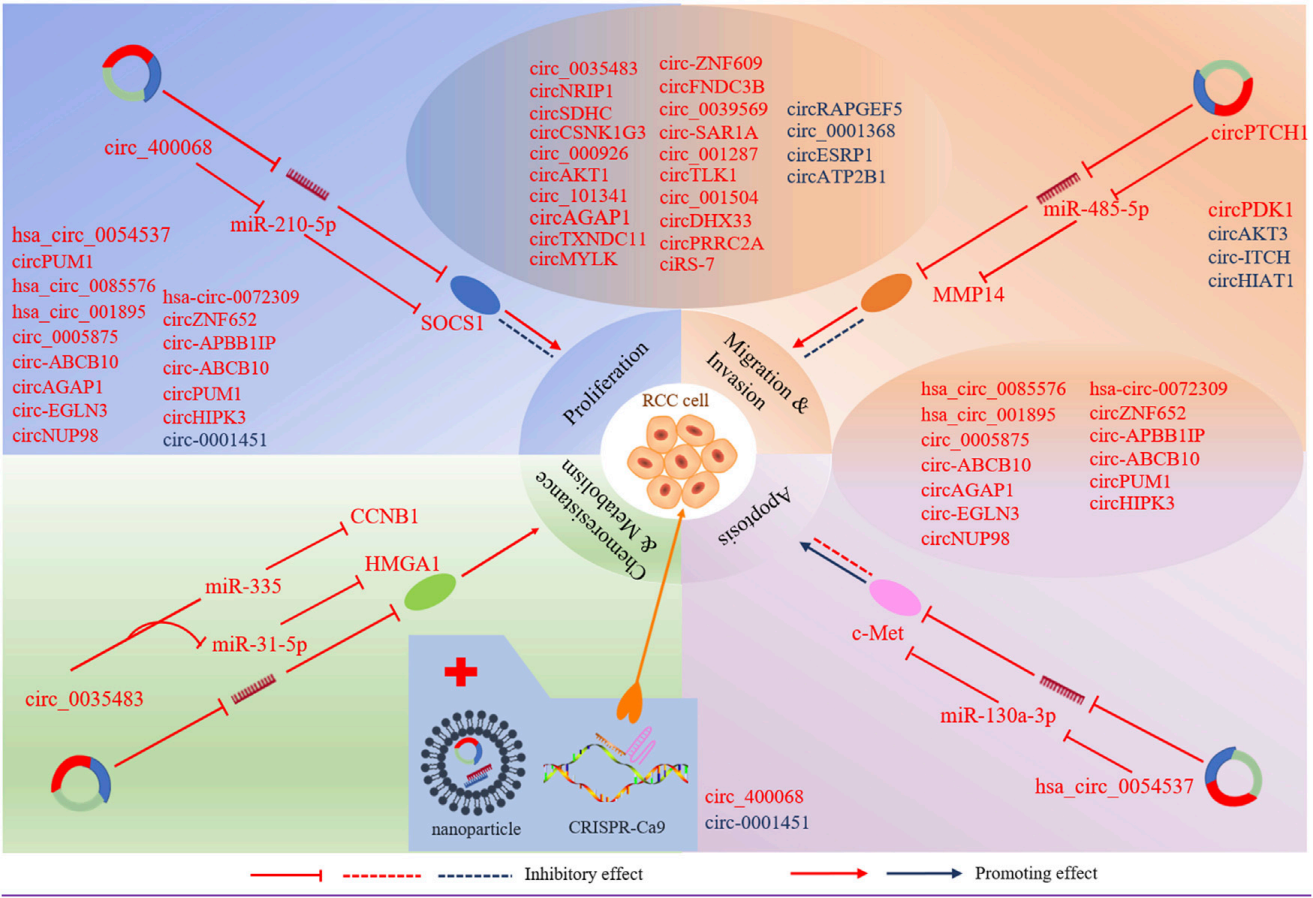
CircRNAs involve in miRNA-associated gene regulatory pathway to regulate RCC cell proliferation, apoptosis, migration, invasion, chemoresistance and metabolism. Selected samples of circRNAs and their genomic targets are exhibited for tumour progression. CircRNAs in red represent up-regulated in RCC, and circRNAs in blue represent downregulated in RCC. Nanoparticles act as delivery vehicles for siRNAs and circRNA expression vectors provide a circRNA -based therapeutic strategy for RCC. CRISPR/cas9 -mediated knockout may be applied to RCC treatment.

The aberrant circRNAs commonly exist in RCC (Table1), regulating cell proliferation, apoptosis, migration, invasion and gemcitabine chemoresistance via cancer-associated signaling pathways (Figure2) and circRNA-miRNA-mRNA interaction networks (Papatsirou et al., 2021) (Figure3).

**TABLE 1:** Dysregulated circRNAs in renal cell carcinoma (RCC).

Name	Expression	Sponge target	Gene/Pathway	Function	Types of RCC tissues and RCC cell lines	PMID
circSDHC	up	miR-127-3p	CDKN3	Promoted cell proliferation and invasion	4 tumors and matched adjacent normal tissue; 498, 786-O, 769P, and Caki-1	33468140
			E2F1 pathway			
circNRIP1	up	miR-505	AMPK and PI3K/AKT/mTOR pathways	Promoted cell proliferation and migration	25 pairs of RCC tissues and the nontumor tissues; ACHN, and CAKI-1	31692056
circFNDC3B	up	miR-99a	JAK1/STAT3 and MEK/ERK signaling pathways	Enhanced proliferation and migration	25 pairs of RCC tissues and adjacent tissues; ACHN and CAKI-1	31637704
circZNF652	up	miR-205	the Ras/Raf/MEK/ERK and JAK1/STAT3 signaling pathways	Increased cells proliferation and EMT, and inhibit apoptosis	22 pairs of Clinical renal carcinoma tissues and corresponding normal tissues	32070139
circ-APBB1IP	up	/	ERK1/2 signaling pathway	Promoted cell proliferation, migration, invasion and inhibited apoptosis	14 pairs of tumor tissues and tumor-distant tissues of ccRCC; 786-O and Caki-1 cell lines	32547313
CircPUM1	up	miR-340-5p	FABP7	Promoted cell proliferation, migration, invasion and inhibited apoptosis	50 pairs of ccRCC tissues and adjacent normal kidney tissues of patients undergoing surgery; HK-2, Caki-2, and 786-O cell lines	33472512
			MEK/ERK pathway			
circTXNDC11	up	/	the MAPK/ERK pathway	Promoted cell proliferation and invasion	30 pairs of RCC tissues and adjacent nonmalignant tissues; ACHN, 786-O, A498, Caki1, and Caki2	34308775
hsa-circ-0072309	down	miR-100	PI3K/AKT and mTOR pathways	Inhibited cell proliferation, migration, and invasion, but enhance cell apoptosis	30 pairs of kidney cancer and the normal tissues; CAKI-1 and ACHN	31456425
circ_001287	up	miR-144	CEP55	Promoted cell proliferation, migration, invasion and tumor growth	77cases of RCC Tumor and adjacent normal tissues; A-498, 786-O, CAKI-1 and CAKI-2	33256799
circPCNXL2	up	miR‐153	ZEB2	Promoted cell proliferation, and invasion of RCC	63 pairs of ccRCC tissues and adjacent non-tumor tissues; A498, 786-O, ACHN, and Caki-1)	30488762
circ_0005875	up	miR-145-5p	ZEB2	Increased cell proliferation, migration and invasion	64 pairs of ccRCC tissues and adjacent normal controls; caki-1,769-p, ACHN, A498	33193877
circAGAP1	Up	miR-15a-5p	E2F3	Enhanced the proliferation, migration and invasion, and inhibited apoptotic of RCC	34 pairs of ccRCC tissues and adjacent nontumor tissues; A498, ACHN, CAKI-1 and OS-RC-2	33618745
Circ_0005875	up	miR-502-5p	ETS1	Promoted cell proliferation, migration and invasion, and inhibited apoptosis and cell cycle arrest	37 pairs of RCC tissue and adjacent normal tissues; HK-2, A498, and Caki-1	34407050
circNUP98	up	miR‐567	PRDX3	Promoted cell proliferation, migration and invasion and inhibited apoptosis	78 pairs of RCC tissues and adjacent normal tissues; 786-o, Caki-1, Caki-2, 769-p and A498	32729669
circ-EGLN3	up	miR-1299	IRF7	Promoted cell proliferation, migration and invasion but inhibited apoptosis	80 pairs of RCC specimens and matched nontumor samples; 786-0, ACHN, CAKI‐1, and OSRC2	31904147
circ-EGLN3	up	miR-1224-3p	HMGXB3	Promoted cell proliferation, migration and invasion, and inhibited apoptosis	43 pairs of RCC tissue specimens and matched non-carcinoma	34274607
					Specimens; HK-2 and five RCC cell lines, A498, 786-O, Caki-1, ACHN, and 789-P	
circPRRC2A	up	miR-514a-5p and miR-6776-5p	TRPM3	Increased cell proliferation, migration and invasion, and induced EMT	118 pairs of RCC tumor tissues with matched normal-adjacent renal tissues; A-498, 786-O, 769-p, ACHN, CAKI-1 and CAKI-2	32292503
circPTCH1	up	miR-485-5p	MMP14	Increased cell migration and invasion and induced EMT	39 RCC tissues and matched adjacent normal samples; ACHN, OS-RC-2, A498, 786-O	32929380
circ_001504	up	miR-149	NUCB2	Promoted cell proliferation, migration and invasion	43 paired RCC tissues and adjacent normal tissues; A-498, 786-O, Caki-2, and Caki-1	34274607
hsa_circ_0054537	up	miR-130a-3p	c-Met	Enhanced cell proliferation and inhibited apoptosis	A-498, Caki-1, SW839 and OSRC-2	32464246
CircPDK1	up	miR-377-3P	NOTCH1	Promoted RCC invasion and metastasis	30 pairs of RCC tissues and para tumor tissues; 786–0, 769-P, and ACHN	33173313
circ_000926	up	miR-411	CDH2	Promoted growth, migration, and invasion abilities of cells, as well as EMT and tumor growth	85 pairs of RCC tissues and adjacent normal tissues; 786-O, A498, Caki-1, and ACHN	31476285
circ_101341	up	miR-411	EGLN3	Promoted proliferation, migration and invasion	60 pairs of ccRCC tissues and matched normal tissues; A498, Caki-1 and 786-O	33408523
Circ_0035483	up	miR-31-5p	HMGA1	Promoted proliferation, migration and invasion and glycolysis of RCC cells	60 pairs of RCC tissues and coterminous normal tissues; 786-O and CaKi-1	33531839
circ_0035483	up	miR-335	CCNB1	Promoted autophagy and tumor growth and enhanced gemcitabine resistance in RCC	5 pairs of kidney cancer tissues and adjacent	31492499
					Tissues; TK10 and UO31 cells	
circTLK1	up	miR-136-5p	CBX4	Promoted cell proliferation, migration, and invasion	60 RCC tissues and matched adjacent normal tissues; ACHN, 786-O and 769-P	32503552
circ-ZNF609	up	miR-138-5p	FOXP4	Promoted cell proliferation, migration, and invasion ability	A-498, ACHN, OS-RC-2, 769-P, G-401	30478938
circ-SAR1A	up	miR-382	YBX1	Promoted RCC cells’ growth and invasion	41 pairs of RCC tissues and matched adjacent normal tissues; 786-O, Caki-1, 769-P, ACHN, and A498	32884349
circ_0039569	up	miR-34a-5p	CCL22	Promoted RCC cell proliferation and metastasis	52 pairs of RCC tissues and their adjacent tissues; ACHN, A498, 786-O, 769-P and RCC4	31497210
circMYLK	up	miR-513a-5p	VEGFC	Promoted cell proliferation, migration and invasion	71 pairs of RCC tissues and matched adjacent normal renal tissues ACHN, 786‐O, and Caki-1	32342645
circ_400068	up	miR-210-5p	SOCS1	Promoted cell proliferation and inhibited apoptosis	28 Human kidney tissue and plasma specimens; Caki-1 and Caki-2	33173957
circAKT1	up	miR-338–3p	CAV1	Promoted cell proliferation, colony formation, migration, invasion and EMT	70 pairs of ccRCC tumor tissues and adjacent normal tissues; 786-O, A498, ACHN, Caki-1, and OS-RC-2	32900491
circDHX33	up	miR-489-3p	MEK1	Promoted the proliferation and invasion	RCC cell lines 786-O, Caki1, A498, Caki2, and ACHN	32717723
circTLK1	up	miR-495-3p	CBL	Promoted cell proliferation, migration and invasion	60 RCC tissues and matched adjacent normal tissues; ACHN, 786-O and 769-P	32503552
Hsa_circ_0085576	up	miR-498	YAP1	Promoted cell proliferation, migration and invasion but inhibited apoptosis	76ccRCC tissues and adjacent normal tissues; 786-O, Caki1, A498 and ACHN	32541093
hsa_circ_001895	up	miR-296-5p	SOX12	Promoted cell proliferation, migration and invasion and inhibited cell apoptosis	60 pairs of ccRCC and adjacent noncancer tissues; 786‐O, A498, OS‐RC‐2, 769‐P and ACHN	31782868
circCSNK1G3	up	miR-181b	TIMP3	Promoted cell proliferation, migration and invasion and induce EMT	786-O, Caki-1, A498 and ACHN	33560588
circ-ABCB10	Up	/	/	Promoted cell proliferation, migration and inhibited apoptosis	120 tumor tissue and paired adjacent tissue; A498, Caki-2, ACHN, Hs891.T and Cal-54	31106654
CircHIPK3	up	MiR-485-3p		Promoted proliferation and metastasis and inhibited apoptosis	48 pairs of tissues and adjacent tissues; HK-2, A498, 768-O, and 769-P	32550826
circHIPK3	up	miR-508-3p	CXCL13	Promoted Proliferation, Migration and Invasion	50 paired ccRCC and adjacent paratumor tissues; HK2, A498, 786-O and 769-P	32821115
ciRS-7	up	miR-139-3p	TAGLN	Inhibited cell proliferation, invasion, tumor growth and metastasis	85 pairs of RCC tissues and adjacent non-tumor tissues; 786-O and ACHN	34740354
circ_0001368	down	miR-492	LATS2	Suppressed cell proliferation and invasion	64 tumor tissues and normal renal tissues; 786-O, ACHN, and A498	32428698
Circ_RPL23A	down	miR-1233	ACAT2	Inhibited cell cycle progression, proliferation, migration and invasion but promoted apoptosis in ccRCC cells	60 pairs of ccRCC tissues and normal tissues; HK2, 786-O,769-p, Caki-1, and A498	34036483
CircRAPGEF5	down	miR-27a	TXNIP	Inhibited proliferation, migration and invasion	42 pairs of RCC tissues and matched adjacent normal tissues; 769-P, Caki-1, OSRC-2, and 786-O	31629934
circAKT3	down	miR-296-3p	E-cadherin	Inhibited cell migration and invasion	60 pairs of ccRCC tissues and paired adjacent normal kidney tissues; OSRC-2, Caki-1, SN12-PM6, A498, and SW839	31672157
circ-ITCH	down	miR-106b-5p	PDCD4	Inhibited migration and invasion of ccRCC cells	54 pairs of ccRCC tissues and paired adjacent normal kidney tissues; OSRC-2, A498, SW839, 786-O, Caki-1, and GRC-1	33969128
circHIAT1	down	miR-195-5p/29a-3p/29c-3p	CDC42	Inhibited AR-dependent migration and invasion of ccRCC cell	40 pairs of primary ccRCC and adjacent normal tissues	28089832
circATP2B1	down	miR-204-3p	FN1	Inhibited ERβ-dependent migration and invasion of ccRCC cell	786-O, A498, Caki-1	29490945
circ-0001451	down	/	/	Inhibited proliferation and promote apoptosis	52 pairs of ccRCC tissues and paraneoplastic tissues; OS-RC-2,786-O, OS-RC-1, ACHN, Caki-1	30271486
CircESRP1	down	miR-3942	CTCF	Inhibited RCC cell migration, invasion, EMT, tumor growth, and metastasis	79 paired RCC tissues and adjacent non-tumor tissues; HK2, 786-0, and ACHN	34775467

#### Cancer-Associated Signaling Pathways

Several signaling pathways, such as PI3K/AKT and mTOR, MAP-ERK and AMPK, that play a key role in the development of RCC, have been reported to be associated with dysregulated circRNAs in RCC. For example, the hsa-circ-0072309 exerted anti-tumor effects through inactivating PI3K/AKT and mTOR pathways in the RCC cell lines. Over-production of hsa-circ-0072309 inhibits cell proliferation, migration, and invasion, as well as the PI3K/AKT and mTOR cascade, but enhances cell apoptosis (Chen et al., 2019). However, circNRIP1 plays an oncogenic role in RCC, which activates adenylate-activated protein kinase (AMPK) and PI3K/AKT/mTOR pathways by targeting miR-505 (Dong et al., 2020). In recent years, the Janus kinase 1/signal transducer and activator of transcription 3 (JAK1/STAT3) and extracellular signal-regulated kinase (ERK) kinase (MEK)/ERK signaling pathways have been widely reported to involve in cancer development (Kang et al., 2011; Deng et al., 2018; Gao et al., 2019; Cui et al., 2020). It has also been proved that some circRNAs play vital roles in the development of RCC by affecting these pathways. For example, circFNDC3B plays an oncogenic role in RCC through activation of JAK1/STAT3 and MEK/ERK signaling pathways to enhance cell viability, colony, and migration (Chen et al., 2020b). Similarly, circZNF652 increased proliferation and EMT of RCC cells by stimulating the Ras/Raf/MEK/ERK and JAK1/STAT3 signaling pathways (Zhang and Guo, 2020). In the past year, several more articles have reported that circRNAs regulate similar signaling pathways. Circ-APBB1IP was found to be significantly overexpressed in ccRCC tissues and play a carcinogenic role in RCC by activating ERK1/2 signaling pathway. Knockdown of circ-APBB1IP by siRNA suppressed the proliferation, migration, and invasion and increased the apoptosis of ccRCC cells (Mo et al., 2020). A novel circular RNA circTXNDC11, which is upregulated in RCC patients, was identified to promote ccRCC progression by activating the MAPK/ERK pathway (Yang et al., 2021). Knocking down circTXNDC11 suppressed cell proliferation and invasion *in vitro* and reduced tumor growth *in vivo*, which offered a potential therapeutic target for RCC treatment. Another study revealed that CircPUM1 upregulated FABP7 expression by competitively binding to miR-340-5p, and then activated the MEK/ERK pathway, thus promoting ccRCC progression (Zeng et al., 2021). A limited number of circRNAs have been reported to play roles in RCC- associated signaling pathways, most of which have been shown to exert oncogenic effects. The underlying mechanisms by which circRNAs regulate these signaling pathways remain unclear. Undoubtedly, substantial efforts will be undertaken to reveal the function of circRNAs in the initiation and development of RCC.

#### CircRNA-miRNA-mRNA Networks

In addition, circRNA-miRNA-mRNA interaction networks play important roles in regulating the proliferation and progression of RCC. Up-regulated circRNAs promote tumorigenic functions of RCC cell lines while down-regulated transcripts repress them.

##### Oncogenic CircRNAs

The majority of up-regulated circRNAs in RCC so far have been shown to play a carcinogenic role by acting as miRNA sponges. For instance, hsa_circ_0054537 is functioned as a competitive endogenous RNA to regulate c-Met expression via sponging miR-130a-3p in RCC, thereby enhancing the cell proliferation and inhibiting cell apoptosis (Li et al., 2020a). CircSDHC can also play the same role in RCC through the miR-127-3p/CDKN3/E2F1 axis, thereby leading to RCC malignant progression (Cen et al., 2021). E2F1, one of E2F transcription factors (E2Fs), participates in the development of many different types of cancer (Iwamoto et al., 2004; Lee et al., 2010), including RCC (Ma et al., 2013). E2Fs are a group of transcription factors that play a pivotal role in cellular proliferation, differentiation, and apoptosis (Ertosun et al., 2016). CircAGAP1 has been reported to increase the expression of E2F3, one of the E2F family transcription factors, by sponging miR-15a-5p, thus enhancing the viability, invasion, and inhibiting the apoptosis of ccRCC cells (Lv et al., 2021). ZEB2 is a DNA-binding transcriptional regulator and plays a major role in the epithelial-to-mesenchymal transition (EMT) (Fardi et al., 2019), which plays a key role in tumor invasion and metastasis in multiple cancers (Wang et al., 2017a). CircPCNXL2 was found to promote the expression of ZEB2 by sponging miR-153 (Zhou et al., 2018). Moreover, circPCNXL2 inhibition suppressed the proliferation and invasion of RCC cells. Like circPCNXL2, circ_0005875 was highly expressed in RCC tumors and cell lines and increased ZEB2 expression via sponging miR-145-5p (Lv et al., 2020). According to another study, the circPRRC2A, generated from the PRRC2A gene, was significantly upregulated in RCC. circPRRC2A upregulated TRPM3 by sponging miR-514a-5p and miR-6776-5p, thereby inducing EMT and invasiveness in patients with RCC (Li et al., 2020b). Extracellular matrix degradation is an important mechanism for tumor invasion and metastasis. Matrix metalloproteinases (MMPs) are zinc-containing endopeptidases that are essential to the degradation of extracellular matrix proteins (Boziki and Grigoriadis, 2018). MMPs permit cells to traverse the ECM to reach distant position, and are therefore closely associated with tumor metastasis (Zhao et al., 2018). CircPTCH1 was found to promote RCC metastasis via sponging the miR-485-5p to upregulate MMP14, a member of the matrix metalloproteinases (MMPs) family (Liu et al., 2020a). Circ_0005875 was identified to promote RCC cell proliferation, migration, and invasion, and inhibit apoptosis and cell cycle arrest by sponging miR-502-5p to upregulate ETS1 (Luo et al., 2021), which might transactive matrix-degrading protease genes (Bolon et al., 1995). These studies suggest that circRNAs can promote RCC metastasis by inducing EMT and the degradation of extracellular matrix. Cell proliferation requires increased uptake of nutrients, and it has been reported that aerobic glycolysis can meet the metabolic requirements of cell proliferation (Lunt and Vander Heiden, 2011). Circ_0035483 has been reported to induce glycolysis of RCC cells through miR-31-5p/HMGA1T axis. The glucose consumption and lactate production in RCC cells were inhibited after circ_0035483 was downregulated, suggesting that circ_0035483 promoted glycolytic metabolism in RCC (Liu et al., 2021). CircRNAs may play a role in angiogenesis, which is essential for tumor cell survival and aggressiveness of RCC. Vascular endothelial growth factor (VEGF), a marker gene of angiogenesis, plays a key role in inducing angiogenesis during tumour growth and metastasis. CircPRRC2A has been reported to induce angiogenesis to promote RCC metastasis by increasing the level of VEGFA (Li et al., 2020b), which is one of the most potent inducers of angiogenesis during tumour growth and metastasis (Tischer et al., 1989). Additionally, Li et al. (2020c) identified that circTLK1 plays an oncogenic role in RCC through the miR-136-5p/CBX4 axis. Moreover, CBX4 expression was positively correlated with VEGFA expression in RCC tissues. Several other regulatory cascades with similar function are as follows, circZNF609/miR-138-5p/FOXP4 (Xiong et al., 2019), circ_001842/miR-502-5p/SLC39A14 (Zhang et al., 2016b), circ-SAR1A/miR-382/YBX1 (Zhao et al., 2020), circ_0039569/miR-34a-5p/CCL22 (Jin et al., 2019), circMYLK/miR-513a-5p/VEGFC (Li et al., 2020d), circ_400068/miR-210-5p/SOCS1 (Xiao and Shi, 2020), circAKT1/miR-338–3p/CAV1 (Zhu et al., 2020), CircPDK1/miR-377-3P/NOTCH1 (Huang et al., 2020), circDHX33/miR-489-3p/MEK1 (Wang et al., 2020b), circ_000926/miR-411/CDH2 (Zhang et al., 2019), circTLK1/miR-495-3p/CBL (Lei et al., 2021), circ_001287/miR-144/CEP55 (Feng et al., 2020), circ_001504/miR-149/NUCB2 (Xin et al., 2021). Hsa_circ_0085576/miR-498/YAP1 (Liu et al., 2020b) and hsa_circ_001895/miR-296-5p/SOX12 (Chen et al., 2020c), circNUP98/miR‐567/PRDX3 (Yu et al., 2020), circ-EGLN3/miR-1299/IRF7 (Lin and Cai, 2020), circ-EGNL3/miR-1224-3p/HMGXB3 (Zhang et al., 2021), circ_101341/miR-411/EGNL3 (Yue et al., 2020), circHIPK3miR-508-3p/CXCL13 (Han et al., 2020).

Yet it’s worth noting that some circRNAs can function as miRNA “reservoirs” to regulate the progression of renal cell carcinoma, which is different from the classical function of circRNAs as a “miRNA sponge”. For example, circCSNK1G3 can promote cell proliferation, migration, and invasiveness of RCC cells via miR-181b/TIMP3 axis. In this research, circCSNK1G3 has a positive regulatory effect on miR-181b, which inhibits the expression of tumor suppressor gene TIMP3, resulting in tumor growth and metastasis in RCC (Li et al., 2021a). Another study reported that circATP2B1 enhanced ccRCC cell invasion by increasing miR-204-3p stability to inhibit FN1 (Han et al., 2018). Previous studies have focused on circRNAs’ function as miRNAs sponges. However, several circRNAs can work as miRNA reservoirs to positively regulate miRNAs, suggesting that circRNAs can regulate miRNAs’ availability and function in a different way.

##### Tumor Suppressor CircRNAs

In addition, circRNAs also have tumor suppressive effect in RCC. For instance, circESRP1 is poor expressed in cancer cells and kidney cancer tissues and acts as a suppressor of tumor through miR-3942/CTCF axis (Gong et al., 2021). CTCF also specifically promote the circESRP1 transcript expression and form a positive feedback loop. Overexpression of circESRP1 inhibits clear cell renal cell carcinoma progression by suppressing c-Myc-mediated EMT pathway. In another study, circ_0001368 suppressed renal cells proliferation and invasion through sponging miR-492 to upregulate tumor suppressor geneLATS2 (Chen et al., 2020d). Moreover, a recent study showed that circ_RPL23A exerted its anti-tumor effect by up-regulating the suppressor gene ACAT2 through competitively binding miR-1233 (Cheng et al., 2021). ACAT2 is an enzyme involved in lipid metabolism, which has been reported to be inversely correlated with the prognosis of ccRCC patients, whereas the underlying mechanism of ACAT2 in ccRCC remains unclear (Zhao et al., 2016). Whether ACAT2 mediates cellular metabolic reprogramming to participate in the regulation of ccRCC progression needs further investigation. Other regulatory cascades have been reported to act as tumor suppressors in RCC included CircRAPGEF5/miR‐27a/TXNIP (Chen et al., 2020e), circAKT3/miR-296-3p/E-cadherin (Xue et al., 2019), and circ-ITCH/miR-106b-5p/PDCD4 (Gao et al., 2021). Like circCSNK1G3, circHIAT1 was identified to inhibit AR-dependent migration and invasion of ccRCC cells by serving as a miRNA “reservoir”. This study revealed that circHIAT1 could increase miR-195-5p/29a-3p/29c-3p activity to inhibit CDC42 expression, thereby suppressing ccRCC progression (Wang et al., 2017b).

Overall, these studies illustrate that circRNAs could act as tumor suppressors or oncogenes to regulate the occurrence and development of RCC, which provide perspectives for the future clinical significances of circRNAs as therapeutic targets and treatment strategies.

### Relationships Between CircRNAs Levels and Clinicopathologic Characteristics in RCC

It has been reported that circRNAs were significantly correlated with many clinicopathological features of renal cell carcinoma, including tumor size, grade, stage, lymph node metastasis (LNM), number of tumors, distant metastasis and recurrence. For instance, circPTCH1 is upregulated in RCC cell lines and tumor samples, and higher levels of circPTCH1 are significantly correlated with advanced Fuhrman grade and greater risk of metastases (Liu et al., 2020a). According to Li et al. (2020b), the circPRRC2A is upregulated in RCC tissues, and its levels are positively correlated with the larger tumor size of RCC. Clinically, high levels of circESRP1 are negatively associated with the advanced tumor size, TNM stage and distant metastasis of ccRCC (Gong et al., 2021). Feng et al. (2020) observed that circ_001287 is highly expressed in RCC tissues and cells, and its levels are strongly correlated with the pathological grade, lymph node, tumor size, tumor node metastasis (TNM) stage, and distant metastasis of RCC patients. Hsa_circ_0085576 has also been reported to be upregulated in ccRCC tissues and cell lines, and its levels are positively correlated with the clinical stage, tumor stage, and distant metastasis (Liu et al., 2020b). Zhao et al. (2020) demonstrated that circ-SAR1A is significantly overexpressed in RCC tissues and cell lines and that its levels are correlated to advanced Fuhrman grade, and lymph node metastasis in RCC patients. CircHIPK3 has been shown to be upregulated in ccRCC tissues and cell lines, and its levels are closely associated with a high TNM grade, lymph node metastasis, distant metastasis, bigger tumor size, and higher Fuhrman grade (Han et al., 2020). Similarly, hsa_circ_0005875 expression levels are significantly associated with tumor size, pathological TNM stage, histological differentiation, and lymphatic metastasis (Lv et al., 2020). Furthermore, the expression levels of circPDK1 expression are significantly increased in RCC, and its levels are positively correlated with lymph node metastasis and distant metastasis of RCC (Huang et al., 2020). The current data showed that the high expression of circAKT1 (Zhu et al., 2020) and circTXNDC11 (Yang et al., 2021) are positively associated with TNM stage, lymph node metastasis. In addition, other circRNAs, such as circSDHC (Cen et al., 2021), circMYLK (Li et al., 2020d), circNUP98 (Yu et al., 2020), circ_001842 (Zeng et al., 2020), circ-ABCB10 (Huang et al., 2019), Hsa_circ_0001451 (Wang et al., 2018), circDHX33 (Wang et al., 2020b), ciRS-7 (Mao et al., 2021), Hsa_circ_001895 (Chen et al., 2020c), circAGAP1 (Lv et al., 2021), circ-AKT3 (Xue et al., 2019), and circ_0001368 (Chen et al., 2020d), have also been proved to be associated with various clinicopathologic characteristics in RCC (Table 2).

**TABLE 2:** Utility of circRNAs for the clinical management of RCC.

circRNA name	Clinical sample	Diagnostic	Prognostic			Clinicopathologic characteristics	PMID
			OS	DFS	PFS		
hsa_circ_0001451	tissue	√					30271486
circHIPK3	tissue	√					32550826
circNOX4	tissue	√					31575051
cir-cRHOBTB3	tissue	√					31575051
circEGLN3	tissue	√					31575051
circEGLN3	tissue		√				33946584
circEHD2	tissue		√		√		33946584
circNETO2	tissue		√		√		33946584
circPRRC2A	tissue		√		√	tumor size	32292503
circTLK1	tissue		√	√		metastasis	32503552
circNUP98	tissue		√	√		TNM stage	32729669
hsa_circ_0085576	tissue		√	√		clinical stage, tumor stage and distant metastasis	32541093
CircRAPGEF5	tissue		√			tumor	31629934
						size, advanced TNM stage and distant metastasis	
circ-ABCB10	tissue		√			pathological grade and TNM stage	31106654
circHIPK3	tissue		√			TNM grade, lymph node metastasis, distant metastasis, tumor size and Fuhrman grade	32821115
circ_101341	tissue		√				33408523
circ_001842	tissue		√			lymph node metastasis	32729666
Hsa_circ_0001451	tissue		√			clinical stage, tumor stage, lymph node, and metastasis	30271486
hsa_circ_001895	tissue		√			TNM stage	31782868
circSDHC	tissue		√			TNM stage	33468140
circPTCH1	tissue		√			advanced Fuhrman grade and greater risk of metastases	32929380
ciRS-7	tissue		√			tumor size, high Fuhrman grade	34740354
circ_001287	tissue					pathological grade, lymph node, tumor size, tumor node metastasis (TNM) stage and distant metastasis	33256799
hsa_circ_0005875	tissue					tumor size, pathological TNM stage, histological differentiation, and lymphatic metastasis	33193877
circPDK1	tissue					lymph node metastasis and distant metastasis	33173313
circMYLK	tissue					tumour size, distant metastasis	32342645
circakt1	tissue					TNM stage, lymph node metastasis	32900491
circTXNDC11	tissue					TNM stage, lymph node metastasis	34308775
circDHX33	tissue					TNM stage and metastasis	32717723
circAGAP1	tissue					tumor size, nuclear grade and clinical stage	33618745
circ-akt3	tissue					Fuhrman grade	31672157
circ_0001368	tissue					T stage and lymph node metastasis	32428698
CircESRP1	tissue					the advanced tumor size, TNM stage and distant metastasis	34775467

### CircRNAs as Diagnostic and Prognostic Biomarkers for RCC

Since circRNAs are very stable and conserved molecules, along with their cell-type-specific and tissue-specific expression patterns, reflecting their potentials as novel biomarkers. Early diagnosis and accurate evaluation of the prognosis of RCC are vital for improving treatment efficacy and reducing the mortality of patients with RCC. Therefore, specific biomarkers are urgently needed for the early diagnosis of primary patients and early identification of the local recurrence or distant metastasis after surgical resection in RCC.

#### CircRNAs as Diagnostic Biomarkers for RCC

Increasing studies identified that the expression of circRNAs shows disease specificity and clinical relevance. For instance, the area under the receiver operating characteristic curve (AUC-ROC) of hsa_circ_0001451 was 0.704 for ccRCC diagnosis, with sensitivity and specificity of 0.755 and 0.608, respectively (Wang et al., 2018). Additionally, according to receiver operating characteristic curve analysis, circHIPK3 was a valuable diagnosis biomarker with AUC of 0.95322 in ccRCC (Han et al., 2020). What’s more, a study showed that the diagnostic value of circRNAs combined with the linear transcripts was higher than that of individual circRNAs. For instance, three circRNAs (circEGLN3, circNOX4, and circRHOBTB3) were identified potential diagnostic biomarkers (Franz et al., 2019). The AUC-ROC of circNOX4 and circRHOBTB3 in RCC tissues were 0.81 and 0.82, respectively, while circEGLN3 showed more reliable diagnostic value with AUC-ROC of 0.98. Further, the combined detection of circEGLN3 and linEGLN3 increased the AUC-ROC to 0.99, with 95% sensitivity and 99% specificity.

#### CircRNAs as Prognostic Biomarkers for RCC

Additionally, some circRNAs play an important role as a prognostic biomarker, such as overall survival (OS), disease-free survival (DFS), and progression-free survival (PFS). A study indicated that high circPRRC2A expression was an independent risk factor of worse OS and poorer metastasis-free survival (Li et al., 2020b). Studies have proved that RCC patients with high expression of circTLK1 (Li et al., 2020c), circNUP98 (Yu et al., 2020), and hsa_circ_0085576 (Liu et al., 2020b) had a lower OS and DFS rate. In contrast, circRAPGEF5 expression was decreased in RCC and its downregulation was significantly associated with poor OS and relapse-free survival (RFS) in patients (Chen et al., 2020e). Studies have shown that high circEHD2 and low circNETO2 levels were an independent predictor of a shortened progression-free survival, cancer-specific survival, and overall survival in patients with ccRCC undergoing nephrectomy (Frey et al., 2021). Kaplan–Meier survival curve revealed that the overall survival of ccRCC patients with high circ_101341 expression was always lower than with low circ_101341 expression (Yue et al., 2020). Furthermore, the OS was worse in ccRCC patients with high tumor tissue circ-ABCB10 expression compared with ccRCC patients with low expression of circ-ABCB10 in tumor tissue (Huang et al., 2019). Similarly, the patient survival rate among the group with high circ_001842 expression was found to be lower than those with low circ_001842 expression level, indicating a positive correlation between circ_001842 and the degree of RCC (Zeng et al., 2020). In addition, other circRNAs, such as Hsa_circ_0001451 (Wang et al., 2018), hsa_circ_001895 (Chen et al., 2020c), circ‐EGLN3 (Lin and Cai, 2020), circHIPK3 (Han et al., 2020), circSDHC (Cen et al., 2021), ciRS-7 (Mao et al., 2021), and circPTCH1 (Liu et al., 2020a) are also significantly correlated with worse OS of patients with ccRCC (Table 2).

The above examples demonstrated that some circRNAs could be promising biomarkers for the diagnosis and prognosis of RCC. However, the differential expression of these circRNAs in tissues cannot be shown in plasma or serum. Therefore, the clinical application of circRNAs as biomarkers still needs further research.

### Therapeutic Role of CircRNAs in Renal Cancer

As mentioned above, aberrant circRNAs are related to the occurrence and development of tumors by modulating various signaling pathways, which are potential targets for novel drugs in RCC. Overexpression or knockdown of related circRNAs might be an effective intervention strategy for RCC progression. Considering that the PI3K/Akt/mTOR signaling pathway is an important regulator of cell survival and proliferation, targeting circRNAs to inhibit PI3K/Akt/mTOR pathway may be an effective way to treat renal cell carcinoma. Studies have shown that overexpression of hsa-circ-0072309 (Chen et al., 2019) and circNRIP (Dong et al., 2020) could exert antitumor effects by deactivating the PI3K/Akt/mTOR signaling pathway. So far, several methods have been developed to change the expression of circRNAs, including siRNA, CRISPR/cas9 -mediated knockout, and extracellular vesicles and nanoparticles for the delivery of circRNAs (Zhang and Xin, 2018). A recent study suggested that PBAE/si-ciRS-7 nanocomplexes targeting CIRS-7 had a stronger inhibition effect on RCC tumor growth and metastasis, and may be a promising gene therapy strategy for RCC (Mao et al., 2021).

At present, drug resistance is a tremendous obstacle to cancer treatment, which needs to be solved urgently. According to the research, high expression of hsa-circ_0035483 was associated with gemcitabine resistance in RCC (Yan et al., 2019). Gemcitabine is a cytotoxic chemotherapeutic drug, which is a deoxycytidine nucleoside analogue. It has an obvious curative effect on renal cell carcinoma, but drug resistance often appears. Overexpression of hsa-circ_0035483 can promote gemcitabine resistance by upregulating cyclin B1 through sponging miR-335 in RCC (Yan et al., 2019). In addition, as described above, downregulation of hsa-circ_0035483 can inhibit cell glycolytic metabolism (Liu et al., 2021). Glycolytic activity is increased in proliferating cells, which is also used as a target site in the therapy of kidney cancer. Therefore, hsa_circ_0035483 could be a promising target for preventing gemcitabine resistance and affecting glycolytic activity in RCC therapy. The contribution and mechanism of circRNAs in the development of antitumor drug resistance in the context of tumors are still at a nascent stage and have not been fully elucidated.

In addition, circRNAs have an impact on the efficacy of some drugs, such as curcumin. Curcumin could suppress renal carcinoma tumorigenesis *in vitro* and *in vivo*. A study reported that overexpression of circ-FNDC3B may weaken the effects of curcumin on inhibiting proliferation and promoting apoptosis of RCC cell through regulating the miR-138-5p/IGF2 axis, which provides a new perspective for the treatment of renal cell carcinoma (Xue et al., 2021).

As circRNAs’ regulatory roles in cancer are gradually being unveiled, circRNAs might be developed as novel effective therapeutic targets. The real clinical application of circRNAs as drugs or targets needs more details. In general, the research on circRNAs as potential therapeutic targets will be a hot spot in the field of oncology.

## Conclusion

In the past decade, the role of circRNAs in carcinogenesis has been widely investigated by researchers. As outlined in this review, a large amount of data indicates that circRNAs are strongly associated with tumorigenesis and progression of RCC. We summarized the differentially expressed circRNAs in RCC and their regulatory pathways on cancer-related biological behaviours. The regulatory role of circRNAs in RCC carcinogenesis and progression reflects their potential therapeutic targets for RCC. CircRNAs are significantly associated with many clinicopathologic characteristics and survival parameters in RCC patients which along with their acceptable diagnostic values render them potential diagnostic and prognostic biomarkers for RCC.

It is worth noting that there are limitations of current research and many challenges remain to be overcome in this field. CircRNAs have a variety of important biological functions, but almost all reported circRNAs in RCC focused on their function as miRNAs sponges. However, it is unclear whether other functions of circRNAs are involved in regulation at the same time, such as regulating gene transcription, functioning as protein decoys, and translating into proteins or peptides, which are worth further research. In addition, circRNAs’ roles in serving as protein decoys and translating into peptide may be promising, which may be involved in the pathogenesis of RCC by regulating the expression of disease-related proteins. CRISPR-Cas13 technique, which can knock out circRNAs without affecting homologous mRNAs (Li et al., 2021b), may become a useful tool for circRNA discovery and functional studies in the future (Koch, 2021). Furthermore, the vast majority of aberrantly expressed circRNAs have not been studied functionally and the effects of circRNAs on the RCC microenvironment and drug resistance have remained elusive until recently. Evidence suggests tumor microenvironment can impact tumor initiation and progression, and drug resistance remains a principal limiting factor to achieve cures in patients with cancer. Therefore, elucidating the role of circRNAs in tumor microenvironment and drug resistance will contribute to the identification of possible targets for therapeutic intervention, which may bring better therapeutic effects. The stability, specificity, and detectability of circRNAs make them promising biomarkers in invasive liquid biopsy. However, most of dysregulated circRNAs in RCC are not specific and sensitive enough for clinical appliance. The selection of optimal time and the cut-off value of circRNAs also requires repeated testing. In the future, more clinical studies should be carried out and more standardized techniques and bioinformatics methods are desired to reliably detect these circRNAs. Since exosomes and nanoparticle have been proved to be good targeted drug delivery tools and could act as delivery vehicles for small interfering RNAs (siRNAs) and circRNA expression vectors (He et al., 2021), gene therapy targeting circRNAs for RCC is promising. However, the efficacy, safety and potential side effects of circRNA-based therapeutic interventions remain unclear, which may be one of the focuses for future research. Therapeutic potential of circRNAs need to be verified in animal models and more research is needed on how to efficiently deliver circRNAs to recipient cells to function without immunologic rejection and with sustained long-term effects. It is becoming clear that increasing exploration into the potential roles of circRNAs will extend our knowledge of pathogenesis mechanisms of RCC and hopefully will be popular topics in the near future. We propose that novel diagnostic and prognosis biomarkers and therapeutic strategies based on circRNAs will serve clinical practice effectively in the future.
